# The Effects of Diet and Exercise on Endogenous Estrogens and Subsequent Breast Cancer Risk in Postmenopausal Women

**DOI:** 10.3389/fendo.2021.732255

**Published:** 2021-09-20

**Authors:** Alleigh G. Wiggs, Justin K. Chandler, Aynur Aktas, Susan J. Sumner, Delisha A. Stewart

**Affiliations:** ^1^Department of Nutrition, University of North Carolina at Chapel Hill, Chapel Hill, NC, United States; ^2^Nutrition Research Institute, University of North Carolina at Chapel Hill, Kannapolis, NC, United States; ^3^Department of Supportive Oncology, Levine Cancer Institute, Atrium Health, Charlotte, NC, United States

**Keywords:** diet, exercise, estrogen, metabolism, postmenopasal women, breast cancer risk, breast cancer prevention

## Abstract

Endogenous estrogens have been associated with overall breast cancer risk, particularly for postmenopausal women, and ways to reduce these estrogens have served as a primary means to decrease overall risk. This narrative review of clinical studies details how various nutritional and exercise lifestyle interventions have been used to modify estrogen levels and metabolism to provide a protective impact against breast cancer incidence. We also summarized the evidence supporting the efficacy of interventions, outcomes of interest and identified emerging research themes. A systematic PubMed MEDLINE search identified scholarly articles or reviews published between 2000-2020 that contained either a cohort, cross-sectional, or interventional study design and focused on the relationships between diet and/or exercise and overall levels of different forms of estrogen and breast cancer risk and occurrence. Screening and data extraction was undertaken by two researchers. Data synthesis was narrative due to the heterogeneous nature of studies. A total of 1625 titles/abstracts were screened, 198 full texts reviewed; and 43 met eligibility criteria. Of the 43 studies, 28 were randomized controlled trials, and 15 were observational studies. Overall, studies that incorporated both diet and exercise interventions demonstrated better control of detrimental estrogen forms and levels and thus likely represent the best strategies for preventing breast cancer development for postmenopausal women. Some of the strongest associations included weight loss *via* diet and diet + exercise interventions, reducing alcohol consumption, and consuming a varied dietary pattern, similar to the Mediterranean diet. More research should be done on the effects of specific nutritional components on endogenous estrogen levels to understand the effect that the components have on their own and in combination within the diet.

## Introduction

Breast cancer is the second most common form of cancer in women, with approximately 255,000 new cases diagnosed in the U.S. annually, resulting in about 42,000 deaths where Black women die at a higher rate compared to White women (1). Several factors increase the risk for a breast cancer diagnosis, such as increasing age, especially after 50 years, genetic abnormalities in the BRCA1 or BRCA2 genes, having dense breast tissue, having children after the age of 30, and no history of breastfeeding. In addition, other risk factors have an association with estrogen exposure within the body, such as the early onset of menstruation (<12 years) or late menopause (>55 years), using hormone replacement therapy, and having a body mass index (BMI) greater than 30 (2). While some of these risk factors are not modifiable, evidence suggests that certain factors may be altered through diet and exercise, such as androgen and parent estrogen levels, metabolite levels, and certain metabolite ratios such as the 2-hydroxyestrone/parent ratio and the 2/16α-hydroxyestrone ratio.

Estrogen and its metabolites have been an important topic of breast cancer research. The main estrogens circulating in the body are estrone, estriol, and estradiol, with estradiol being the most biologically active (3). Studies have shown that increased endogenous estrogen levels lead to an overall increase in breast cancer risk. For example, in postmenopausal women, the predominant production pathway of estrogens involves the conversion of androstenedione to estrone catalyzed through the enzyme aromatase (4). In addition to total parent estrogen levels, estrone and estradiol may be irreversibly metabolized through cytochrome P450 enzymes, primarily found in the liver (4). Several of these metabolites have been shown to affect overall breast cancer risk (4). The 2-hydroxylation pathway of estradiol and estrone that includes 2-hydroxyestrone, 2-hydroxyestradiol, 2-methoxyestrone, and 2-methoxyestradiol has been of interest in recent years. The cytochrome P450 enzymes CYP1A1, CYP1B1, and CYP1A2 help catalyze these pathways, generating end products that are less potent than the parent estrogen. These 2-hydroxylated estrogen metabolites have demonstrated a protective effect against breast cancer development (4). The 16-hydroxylated metabolites, specifically 16a-hydroxyestrone, have been shown to promote cancer cell growth and proliferation of breast cells in animal models.

In 2017, Sampson et al. took serum or urine samples from four published studies and processed the samples with a highly specific liquid chromatography-tandem mass spectrometry (LC-MS/MS) method (5). These studies included the Prostate, Lung, Colorectal, and Ovarian Cancer Screening Trial (PLCO), the Columbia Missouri Serum Bank NCI’s Biological Markers Project, The Breast and Bone Follow-up to the Fracture Intervention Trial (B-FIT), and the Shanghai Women’s Study, where each followed breast cancer incidence. In each study, increased levels of parent estrogens (i.e., estradiol and estrone) were associated with an increased risk of breast cancer incidence. In addition, participants who had a higher proportion of estrogen metabolism occurring in the 2-hydroxylation pathway had a lower breast cancer risk than women with higher parent estrogen levels or metabolism by the 16-hydroxylation pathway. The study also found a 50% decrease in breast cancer risk for women with total parent estrogen levels but a high proportion being metabolized in the 2-hydroxylation pathway. Kensler and Zhang also illustrated that an increased level of parent estrogens led to an increased risk of breast cancer in epidemiological studies (4, 6). These papers have opened a door for further studies to investigate what factors may influence overall parent estrogen levels, the 2-hydroxy metabolite to parent estrogen ratio, and the 2/16-hydroxy metabolite ratio with subsequent breast cancer risk.

We, therefore, undertook a narrative review and synthesis to evaluate the influence of diet and exercise interventions on overall estrogen levels, estradiol levels, sex hormone-binding globulin, or the 2/16α-hydroxyestrone ratio and subsequent breast cancer risk in postmenopausal women. Although we discuss the importance of 2/16 hydroxy-estrone, measurement of this metabolite requires more sensitive and newer analytical methods for detection, and many of the studies included in the review did not look at it. Our secondary objective was to summarize the type of interventions and the evidence supporting efficacy, outcomes of interest and identify emerging research themes. 

## Methods

### Search Strategy and Selection Criteria

A systematic search strategy was developed by the research team and conducted through the Health Sciences Library at the University of North Carolina at Chapel Hill. A PubMed MEDLINE search conducted using two sets of search terms/phrases: “estrogen AND diet* OR nutrition And “breast cancer” AND postmenopausal” and “estrogen AND exercise OR “physical activity” AND “breast cancer” AND postmenopausal”. All initial studies from the two searches were uploaded to Covidence systematic review software (Veritas Health Innovation, Melbourne, Australia). Duplicate citations were eliminated during the screening process. All titles and abstracts retrieved from the literature search were screened independently (AW, JC). Potentially eligible studies were retrieved in full and reviewed independently (AW, JC). Discrepancies were resolved by consensus between two researchers. Finally, a reference hand search was done of all included articles and relevant systematic reviews for additional studies.

Inclusion criteria for scholarly articles or reviews consisted of being published between 2000-2020, containing a cohort, cross-sectional, or interventional study focusing on the relationship amongst diet and/or exercise and overall estrogen levels, estradiol levels, sex hormone binding globulin, or the 2/16α-hydroxyestrone ratio, and primarily focused on studies analyzing postmenopausal women not classified as breast cancer patients or survivors. The initial exclusion criterion was being published before 2000. In addition, articles were excluded if they focused solely on pre-menopausal women or breast cancer patients or survivors or if the study focused on a diet and or exercise intervention without investigating hormones. Case reports and conference abstracts were not included. Language was limited to English only.

Each of the manuscripts reported herein were initially summarized (AW), then reviewed for accuracy of information (JC). The data from the included studies was summarized in **Table 1**. Demographics compiled for each study **(** **Table 2** **)** are reviewed herein and used for comparative determinants across studies. **Tables 3**, **4** and **5** each summarize the impact on breast cancer risk and estrogens level changes determined to be increased or decreased with the reported statistical parameters (p-Value or confidence interval) based on the intervention type for included studies using dietary interventions (**Table 3**), exercise interventions (**Table 4**) or dual interventions combining diet and exercise (**Table 5**).

**Table 1:** Summary of studies included for review.

Intervention Diet &/Exercise	Intervention Focus	Study Design	Number of participants (analysis)	Sample type	Primary outcomes	Estrone (E1)	Estradiol (E2)	2/16 α-hydroxy-estrone ratio	SHBG	Ref.
Diet	Alcohol	O	1093	Blood	Levels of DHEAS, E1, E2 were higher in women that consumed more alcohol.	↑	↑			(7)
Diet	Alcohol	O	1291	Blood	Androgens and E1 concentration positively associated with alcohol consumption. Individuals consuming more than 25g/day as opposed to non-consumers had a 20% higher concentration in DHEAS, free testosterone, and estrone, while SHBG were approximately 15% lower.	↑	↑		↓	(8)
Diet	Alcohol	E	53	Blood	E1 levels increased with increased alcohol consumption		↑			(9)
Diet	Animal Products	O	766	Blood	Total red and fresh meat consumption were inversely related with SHBG, higher consumptions of dairy products were associated with increased levels of free and total E2.		↑		↓	(10)
Diet	Overall Diet	O	653 (blood) 27488 (analytical)	Blood,	Women diagnosed with breast cancer were more often in the highest tertiles of the ERDP. The strongest correlates with unconjugated E2 were non-whole/refined grains, cheese, yogurt, and franks/luncheon meats. Only intakes for non-whole/refined grains and cheese were significantly correlated with the 2/16 ratio.					(11)
Diet	DHA	E	25	Urine	DHA supplementation did not have a statistically significant effect on estrogen levels.	=	=			(12)
Diet	Fat intake	O	324	Blood	There was a positive association between estrone levels and total fat intake. DHEAS levels were significantly associated with the percentage of energy from total fat, saturated fat, monounsaturated fat, and polyunsaturated fat.	↑				(13)
Diet	Fat intake	E	46	Urine	The low-fat diet had an increase in E1, and E1+E2+E3, other metabolites were not significantly different between the three diets.	↑ (low fat)				(14)
Diet	Fat intake	E	17	Urine	There was a statistically significant association between the high fat diet and E2 levels.		↑ (high fat)			(15)
Diet	Fat Intake	O	37	Urine	A low-fat high fiber diet was associated with higher 2/16 urinary metabolite ratios.			↑ (low fat high fiber)		(16)
Diet	Flaxseed	E	99	Blood	Women in the intervention group had an increase in 2-hydroxyestrone levels and the 2/16α hydroxyestrone ratio.			↑		(17)
Diet	Flaxseed	E	30	Urine	There was an increase in the 2-hydroxyestrone levels of women in the intervention group that was statistically significant, but changes in the 2/16 ratio were not statistically significant.			=		(18)
Diet	Flaxseed	E	43	Urine	The 2/16α-hydroxyestrone ratio decreased in the urine samples, 16a-hydroxyestrone ratio levels increased.			↓		(19)
Diet	Grape Seed Extract	E	39	Blood	The supplementation did not decrease plasma estrogens.					(20)
Diet	Grapefruit	E	59	Blood	E1S levels increased hours after consuming the grapefruit, but returned back to normal. E1 decreased hours after consuming but returned to normal after 10 hours.	↑(E1S) ↓(E1)				(21)
Diet	Grapefruit	O	876	Blood	There was a positive association between grapefruit intake and increased SHBG				↑	(22)
Diet	Green Tea	E	937	Blood, Urine	There were not reductions in sex hormone levels in the women in the intervention group.					(23)
Diet	Mediterranean Diet	E	106	Urine	The reduction of total estrogen levels in the intervention group was statistically significant. This reduction was due to metabolites instead of E1, or E2 reductions.					(24)
Diet	Overall Diet	E	50	Blood, Urine	Changes in body weight were significantly associated with changes in SHBG.				↑	(25)
Diet	Overall Diet	O	205	Blood	A western dietary pattern, often characterized with increased consumption of red meats, chicken, and eggs, was associated with higher levels of estradiol in the study.		↑			(26)
Diet	Pomegranate	E	64	Blood	When stratified by BMI, normal weight women in the intervention arm had a statistically significant decrease in serum estrone and testosterone levels, but not in women that were overweight or obese by the BMI scale.	↓ (normal weight women)				(27)
Diet	Soy	E	60	Blood	No statistically significant changes in the experimental group.					(28)
Diet	Soy	E	18	Urine	The 2/16α hydroxyestrone ratio was increased by the low iso (65+/- 11 mg soy isoflavones per day) diet.			↑		(29)
Diet	Soy	O	144	Blood	Women who consumed greater than the median (32.2 pg/mL) had E1 levels that were lower than those below the median.	↓				(30)
Diet	Soy	O	167	Blood	No statistically significant changes.					(31)
Diet	Soy	E	57	Blood	There were no associations between sex hormones and the diet interventions between the arms. There were some statistically significant changes in testosterone, but not between the three groups.					(32)
Diet	Soy	E	20	Blood	There was an increase in SHBG from baseline to the end of the ten weeks.				↑	(33)
Diet	Soy	E	74	Blood, Urine	No statistically significant changes.					(34)
Diet + Exercise	Weight Loss	E	421	Blood	Estrone decreased in all interventional arms, estradiol decreased in the diet and diet + exercise arms. SHBG increased significantly in the diet and diet + exercise arm.	↓	↓ (diet, diet + exercise)		↑ (diet, diet + exercise)	(35)
Diet + Exercise	Weight Loss	E	7	Blood, Breast Fluid	Reductions of estradiol in ductal fluid and the blood sample were found at 12 weeks.		↓			(36)
Diet + Exercise	Weight Loss	E	22	Blood	SHBG increased in both groups, 39% in the HRT group and 42% in the non HRT group.				↑	(37)
Diet + Exercise	Weight Loss	E	243	Blood	Overall, participants in the intervention arms had a decrease in all measured hormones and an increase in SHBG.	↓	↓		↑	(38)
Exercise	Activity Level	O	2082	Blood	Increased physical activity was associated with lower levels of estradiol.		↓			(39)
Exercise	Activity Level	O	542	Urine	Higher average activity levels was associated with overall lower levels of parent estrogens.	↓	↓			(40)
Exercise	Activity Level	E	173	Blood	Exercisers had a decrease in estrone at the 3 and 12 month time frames, and an increase in SHBG. At 3 and 12 months, concentrations only changed in exercisers that lost at least.5%. body fat.	↓				(41)
Exercise	Activity Level	O	1804	Blood	Sitting for at least 10 hours a day is correlated with increased unconjugated estrone and estradiol.	↑	↑			(42)
Exercise	Activity Level	O	806	Blood	Inverse association between usual physical activity and free estradiol levels, and a positive association with SHBG.		↓		↑	(43)
Exercise	Exercise Type	E	35	Blood	No effect on estrone or estradiol.					(36)
Exercise	Training	E	163	Urine	Overall, there were no changes between the groups in their estrogen metabolite ratio.					(44)
Exercise	Training	E	320	Blood	Before weight change was adjusted for, total estradiol, free estradiol, and SHBG changed in the exercise intervention group.		↓		↑	(45)
Exercise	Training	E	400	Blood	The exercise prescription was associated with decreases in E2, estrone, and free E2 and increases in SHBG, but the differences between the high and moderate prescriptions were not statistically significant.	↓	↓			(35)
Exercise	Training	E	307	Blood	While participants in the intervention group had an overall decrease in estradiol levels, there were no statistically significant changes in estrogen metabolism pathways.		↓			(37)
Exercise	BMI EMM	O	267	Blood	Women with high BMI and low physical activity had the highest levels of levels of estrone and free estradiol.	↑	↑			(46)

Abbreviation for SHBG is sex-hormone binding globulin, DHEAS is dehydroepiandrosterone sulfate. Study designs as O for an observational style study and E for an experimental style study, and Ref. is reference. “↑”, “↓” and “=” stand for "increased" , "decreased" and "no change".

**Table 2:** Demographics and clinical characteristics of the study participants of the eligible studies.

Number of Participants	Mean Age (Standard Deviation)	Race/Ethnicity	Alcohol Consumption (g/day)	Family History of Breast Cancer	Natural Menopause	Surgical (Bilateral oophorectomy/hysterectomy) Induced Menopause	Hormone Use	Mean BMI	Ref.
173	60.65	149 (86%) White	4.3	56 (32.4)		31 (17.9)	73 (42.2) (ever)	30.4	(41)
		7 (4.0%) African American							
		2 (1.2%) Hispanic							
		9 (5.2%) Asian							
		2 (1.2%) Native American							
		3 (1.7%) Other							
1804	62.3	1640 (86.1%) White					868 (48.1) current		(42)
542	60.6		198 current/former (36.5)	38 (7.0)			0 current	27.2	(40)
35	57.2	31 (88.6%) White						33.3	(36)
43	57.3	41 (97.7%) White		7 (16.3)			18 (41.9) ever	25.6	(19)
421	58 (5)	373 (85.4%) White	7.3	153 (35)		117 (24.8)	261 (62%) ever	30.9	(47)
		35 (4.3%) African American							
		12 (2.8%) Hispanic							
		8 (1.7%) Asian							
		11 (4.3%) Other							
99								27.1	(28)
766	61		384 (50) ever		766 (100)		84 (11) ever	27.2 (4.6)	(10)
57	60								(12)
307	61	279 (91%) White						29	(37)
806	60.1 (5.2)		2.9				77 (9.6) ever	26.2 (4.8)	(43)
320	60.9	289 (90%) White	4.7	66 (21) first degree			146 (46) ever	29.2	(45)
243	60			56(23)				29.2	(48)
163	60.7	141 (86.6%) White	Less than 2 drinks/day				0	30.45	(44)
324	57.5 (5.9)		Yes	12 (3.7)				23.3 (3.0)	(13)
937	59.8	916 (97.7) White	3.4	234 (25.0)	805 (85.9)	80 (8.53)	380 (40.5) ever	25.1	(23)
99	60	93 (94%) White	Yes					26	(17)
30	56.6							27.5	(18)
1093	59.5		Yes		625 (57)		154 (14) ever	26.06	(7)
16	58	14 (88%) White					none actively	27	(14)
		1 (6%) African American							
		1 (6.6%) Asian							
940									(22)
2082	69		3.96				116 (13.4)	27.1	(43)
400	59.5	364 (91%) White	2.7	72 (18)		80 (20)	117 (29.3)	29.2	(36)
37	61.7	37 (100%) White	under 2 drinks/day	13 (35)				27.7	(16)
305	56.6		Yes	6 (2)					(26)
22	64.1 (1.7)		No	No			11 (current)	32.1	(38)
267	64.8 (6.9)	210 (78.7%) White	2 servings/week			89 (33)	not in last 3 months	30.1 (6.4)	(46)
		42(15.6%) African American							
		4 (1.5%) Hispanic							
		4 (1.5%) Asian							
		3 (1.1%) Native American							
		3 (1.1%) Other							
60	55.7						0	23.5	(28)
7	55.3 (5.5)	6 (85.7%) White						33.6	(49)
		1 (14.3%) Asian							
653 (blood) 27488 (analytical)	62.4							27.1	(11)
59	57.4		Yes					26.7	(21)
106	48-69					0	0		(24)
53	58.2	39 (74.5%) White	Yes,.9 drinks/day			9 (17.6)	0 current		(9)
		11 (21.6%) African American							
		2 (3.9%) Asian							
25	52.5						0 last 6 months	26.9	(12)
39	65.5						0 last 6 months	13 >30	(20)
17	57					0	0 during study	28	(15)
64	58.3	60 (93.8%) White	48 (yes)	5 (7.8)	64 (100)	0	11 ever	24.7	(27)
20	54.3 (5.7)							27.3	(33)
74	60							28.1	(34)
18	56.9		0 during study		18 (100%)		0 last 6 months	25.2	(29)
57	60								(32)
144	60	144 (100%) Asian							(30)
167									(31)

Abbreviation for BMI is body mass index and Ref. is reference.

**Table 3:** Breast cancer risk and estrogen level changes assessed in dietary intervention studies.

Intervention Focus	Estrone (E1)	Estradiol (E2)	2/16 α-hydroxy-estrone ratio	SHBG	Impact on breast cancer risk	Reported p-Value	Ref.
**Alcohol**	↑	↑			↑	E1(0.001), E2 (0.03)	(7)
**Alcohol**	↑			↓	↑	E1 (0.0001), SHBG (0.03)	(8)
**Alcohol**	↑				↑	30 g/day E1 (0.009)	(9)
**Animal Products**		↑		↓	↑	Total E1 (0.02), Free E1 (0.03)	(10)
						SHBG Total Red Meat (0.04) SHBG Fresh Red Meat (<0.01)	
**DHA**	=	=	=		=		(12)
**Fat intake**	↑				↑	E1 (0.04)	(13)
**Fat Intake**	↑ (low fat)	↑ (low fat)			↑	Total E1 (0.02), E1+E2+E3 (0.02)	(14)
**Fat Intake**		↑ (high fat)				Plasma E2 (0.02)	(15)
**Fat Intake**			↑ (low fat high fiber)		↓	95% CI (−0.46, −0.10),	(16)
**Flaxseed**			↑		↓	95% CI: (1.15-2.06)	(17)
**Flaxseed**			=		=		(18)
**Flaxseed**			↓		↑	2/16*α*-OHE_1_ (0.02)	(19)
**Grape Seed Extract**	=	=			=		(20)
**Grapefruit**	↑(E1S) ↓(E1)				↓	E1 after 4 hours (0.009)	(21)
**Grapefruit**				↑	↓	SHBG (0.03)	(22)
**Green Tea**					=		(23)
**Mediterranean Diet**					=		(24)
**Overall Diet**				↑	↓	SHBG (0.0001)	(25)
**Overall Diet**		↑			↑	E2 CI Western Diet (0.1 – 0.29), Eggs (0.106-0.441), Red Meat (0.01 – 1.01)	(26)
**Pomegranate**	↓ (normal weight women)				↓	E1 (0.05)	(27)
**Soy**					=		(28)
**Soy**			↑		↓	NR	(32)
**Soy**	↓				↓	E1(0.047)	(30)
**Soy**					=		(31)
**Soy**					=		(32)
**Soy**				↑	↓	SBHG (<0.05)	(33)
**Soy**					=		(34)

NR, not reported. “↑”, “↓” and “=” stand for "increased" , "decreased" and "no change".

**Table 4:** Breast cancer risk and estrogen level changes assessed in exercise intervention studies.

Intervention Focus	Estrone (E1)	Estradiol (E2)	2/16 α-hydroxy-estrone ratio	SHBG	Impact on breast cancer risk	Reported p-Value	Ref.
**Activity Level**		↓			↓	E2 (<0.05)	(39)
**Activity Level**	↓	↓			↓	High Activity (0.01)	(40)
**Activity Level**	↓				↓	E1 (0.03)	(41)
**Activity Level**	↑				↑	Sedentary Time E1 (0.03)	(42)
**Activity Level**		↓		↑	↓	E2 (0.045) SHBG (0.05)	(43)
**Exercise Type**					=		(36)
**Training**					=		(44)
**Training**		↓		↑	↓	E2 (0.88 -0.98) SHBG (1.02 – 1.07)	(45)
**Training**		↓			↓	E2 (0.002)	(35)
**Training**		↓			↓	E2 (0.04)	(37)

“↑”, “↓” and “=” stand for "increased" , "decreased" and "no change".

**Table 5:** Breast cancer risk and estrogen level changes assessed in dual intervention studies.

Intervention Focus	Estrone (E1)	Estradiol (E2)	2/16 α-hydroxy-estrone ratio	SHBG	Impact on breast cancer risk	Reported p-Value	Ref.
**Weight Loss**	↓	↓ (diet, diet + exercise)		↑ (diet, diet + exercise)	↓	SHBG (<0.001), E1 (<0.001), E2 (<0.001)	(47)
**Weight Loss**		↓			↓	E2 (−113.1, -10.5)	(49)
**Weight Loss**				↑	↓	SHBG (<0.01)	(38)
**Weight Loss**	↓	↓		↑	↓	E2 (<0.001), SHBG (<0.001)	(48)

“↑” and “↓” stand for "increased" and "decreased".

Studies were systematically selected based on two sets of search terms/phrases the search procedures described below. The search phrases “estrogen AND diet* OR nutrition And “breast cancer” AND postmenopausal” and “estrogen AND exercise OR “physical activity” AND “breast cancer” AND postmenopausal” through PubMed. All initial studies from the 2 searches, which yielded 1159 results were included, then ran through Covidence (www.covidence.org, results returned through https://app.covidence.org/reviews/108352). From there, studies were secondarily screened based on the criteria of interest. Inclusion criteria for scholarly articles or reviews consisted of being published between 2000-2020, containing a cohort, cross-sectional, or interventional study focusing on the relationship amongst diet and/or exercise and overall estrogen levels, estradiol levels, sex hormone binding globulin, or the 2/16α-hydroxyestrone ratio, and primarily focused on studies analyzing postmenopausal women not classified as breast cancer patients or survivors. The initial exclusion criterion was being published before 2000. In addition, articles were excluded if they focused solely on pre-menopausal women or breast cancer patients or survivors or if the study focused on a diet and or exercise intervention without investigating hormones. For our review, the term statistically significant is used when the p-value for reviewed study outcomes was less than.05. The term borderline significant is used when the p-value was described between 0.08-0.05. Each of the manuscripts reported herein were initially summarized by Alleigh Wiggs, then reviewed for accuracy of information by Justin Chandler through the systematic selection processes described in **Tables 1** and **2**.

## Results

### Summary of the Literature Search

**Figure 1:**
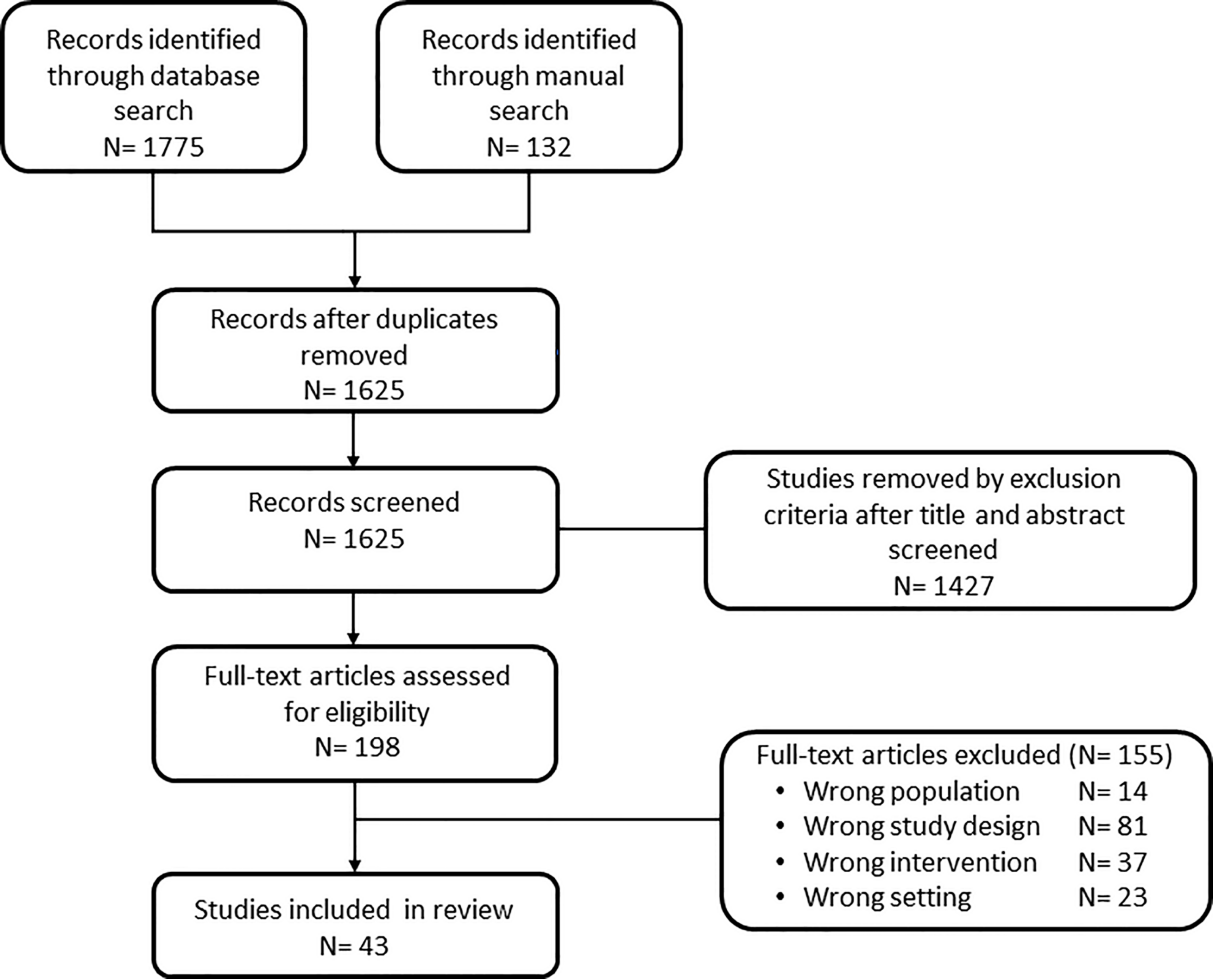
Flowchart of systematic literature search process. N is abbreviation for number.

The literature search process is outlined in **Figure 1**. The database search yielded 1775 records and 132 additional manuscripts were identified through hand search. After removing duplicates, 1625 titles/abstracts were screened, 198 full texts were reviewed following removal based on exclusion criteria; and 43 met inclusion criteria and were included in the review. Of the 50 studies, 28 were randomized controlled trials, defined as an experimental study style in **Table 1**, and 15 were an observational study style.

### Diet

#### Alcohol

The Centers for Disease Control and Prevention (CDC) lists alcohol consumption as a risk factor for developing breast cancer (2). While alcohol may affect the body in various ways, evidence suggests that alcohol consumption may be related to increased estrogen levels. In a cross-sectional study by Onland-Moret et al. in 2005, postmenopausal women from the European Prospective Investigation into Cancer from Utrecht, The Netherlands (Prospect - EPIC) cohort consuming more than 25g of alcohol per day had increased levels of estradiol as opposed to their non-drinking peers (7). In another analysis of the larger EPIC Cohort by Rinaldi et al. in 2006, postmenopausal women consuming more than 25g of alcohol per day had 20% increased levels of free testosterone and estrone, while sex hormone binding globulin (SHBG) values were 15% lower, even when accounting for BMI (8). In a randomized controlled cross-over trial by Dorgan et al. in 2001, women were placed into a 0mg, 15mg, or 30mg of alcohol per day regimen, with calorie intake controlled between the groups. Women consuming the greatest proportion of alcohol, 30 mg/day, had an average of 10.7% increase in their estrone levels, even after age, BMI, race, or years since menopause were accounted for (9).

Interestingly, the Onland-Moret et al. and Rinaldi et al. studies show differing conclusions. Onland-Moret et al. found a significant increase in estradiol, while Rinaldi et al. found a significant increase in estrone (7, 8). These results may be due to analyzing data from different cohorts within the overall EPIC initiative. However, both studies found statistically significant increases in parent estrogens (7, 8).

#### Animal Products

The International Agency for Research on Cancer has classified red meat as “probably carcinogenic to humans” and processed meat as “carcinogenic to humans” based on its links to colon cancer (50). Brinkman et al. in 2010 investigated if meat consumption affected estrogen levels in postmenopausal women in the Melbourne Collaborative Cohort Study. The study found that total red and fresh meat consumption were inversely correlated with SHBG. Furthermore, increased dairy consumption was associated with higher levels of free and total estradiol, with women in the highest quartiles of dairy consumption having 15% higher levels of total estradiol (10). No significant association between serum estrogens and eggs, poultry, fish, eggs, butter, or processed meats were found. By contrast, a 2018 study found a strong correlation between the consumption of processed meats and cheese to unconjugated estradiol levels, while yogurt consumption demonstrated an inverse correlation with estradiol levels (11). Thus, there may be differences in the dairy products consumed and their subsequent effect, especially considering the ability of the dairy product to impact gut microbiota dramatically. Associated foods may also play a role; cheese for example, which is often consumed as part of dishes including pizza and pasta, where these other components possibly contribute to the overall estrogenic effect.

#### Grapefruit

Grapefruit is known to interfere with estrogenic hormone replacement therapy when taken orally, possibly because it inhibits the cytochrome P450 3A4 enzyme, which may have activity in metabolizing parent estrogens (22). If grapefruit acts to decrease enzyme activity, it may subsequently increase estrogen levels in the blood. In a cross-sectional study by Spencer et al. in 2009, increased grapefruit consumption in the EPIC cohort was not associated with an increased risk of breast cancer (22). While there were no associations between grapefruit consumption and estrogen levels, there was a positive relationship between consumption and SHBG. In 2013, Monroe et al. found that grapefruit supplementation was linked to fluctuations in overall estrone sulfate levels several hours after consumption of grapefruit juices and sodas. In their study, estrone sulfate levels were on average 11.4% higher 24 hours after supplementation and remained elevated throughout the two weeks of intervention (21). Although the study found grapefruit juices and sodas caused a change, there were no associations between estrone sulfate and whole grapefruit consumption.

Grapefruit consumption in certain forms or amounts may affect estrogen levels; however, it is not clear that habitual consumption has adverse outcomes. If grapefruit consumption also is associated with an increase in SHBG, as was shown in the EPIC cohort, the increased level of estrone in the body may be rendered less biologically inactive. More studies are needed to identify the exact mechanism by which grapefruit interacts with the cytochrome P450 3A4 enzyme and endogenous estrogens, particularly as the same cytochrome P450 enzymes could be involved in the metabolism of drugs used in the treatment of cancers.

#### Flaxseed

In a 2013 systematic review, Flower et al. found an association between flaxseed consumption and reduced breast cancer risk (51). Flaxseed is a natural source of dietary lignans, fiber, and α-linolenic acid. These components have long been of interest in breast cancer research, and studies have investigated if the mechanism by which flaxseed protects against breast cancer is associated with endogenous estrogen levels. In a study by Sturgeon et al., women consumed 1 tablespoon (7.5 grams) of ground flaxseed for 6 weeks, followed by consuming 2 tablespoons (15 grams) for an additional six weeks. At 12 weeks, there was a borderline significant increase in urinary estradiol (p = 0.07), and a borderline significant decrease in urinary estrone (p = 0.08). In contrast, a statistically significant increase in 16a-hydroxyestrone levels in the urine at 12 weeks and a subsequent reduction in the 2/16α-hydroxyestrone ratio were shown (19). This finding is surprising as 16a-hydroxyestrone is known to be the more biologically active estrogen metabolite. Another study from Laidlaw et al. investigated the effects of a breast health formula, including 20mg of dietary lignans and 400mg of indole-3-carbinol (I3C), a compound found in brassica vegetables, per day. This study contrasted the result of Sturgeon and colleagues, demonstrating increasing levels of urinary 2-hydroxyestrone and the 2/16α-hydroxyestrone ratio; however, enterolactone serum levels showed no significant increase (18). Similarity in the studies was present, though, where Laidlaw et al. did also see an increase in urinary 16a-hydroxyestrone levels in their intervention group, but it was at a nonsignificant threshold (18). In 2019, Chang et al. asked women to consume 2 tablespoons of ground flaxseed every day for seven weeks. Women in this intervention group saw an increase in serum 2-hydroxyestrone levels and an increase in the 2/16α-hydroxyestrone ratio (17). More information is needed to understand the mechanism(s) through which flaxseed may affect estrogen metabolites. Additionally, there may be differences between urinary and serum estrogen levels that need to be addressed. The latter study had a similar protocol in dosage and intervention length to the study conducted by the former (Sturgeon) but found directly contrasting results; in that Chang et al. found an increase in 2-hydroxyestrone and the 2/16α-hydroxyestrone ratio while the former found an increase in 16a-hydroxyestrone levels and no change to the 2/16α-hydroxyestrone ratio.

#### Fat Intake

Many studies over the past 20 years have investigated fat intake and estrogen levels in postmenopausal women. In 2001, Fowke et al. investigated overall dietary intake and estrogen metabolites. With each increase of 10g of saturated fat per day there was a subsequent 0.52 decrease in the 2/16α-hydroxyestrone ratio (16). In a cross-sectional study of Japanese women in 2005, Nagata et al. found a positive association between a 5% increase in total fat intake and estrone levels (13). Additionally, Dehydroepiandrosterone sulfate (DHEAS), a precursor to estradiol, was associated with an increasing percentage of total fat, saturated fat, mono-unsaturated fat, and polyunsaturated fat. However, it should be noted in this cohort, the highest quartile of calories from fat consumption was 28% or more, with 14.8% of the women consuming greater than 30% of their calories from fat (13). The Center for Disease Control and Prevention’s National Center for Health Statistics reports that in 2018, the average intake of fat for women in the United States more than 20 years of age was 35% of daily calories (52). The women in the Nagata et al. study on average consumed 25% of total calories from fat and 6.7% of calories from saturated fat (13), which is consistent with values recommended by the World Health Organization (WHO) (53). This is notable as the fat intake seen in the Nagata et al. study differs from the average American diet, and results between studies may differ due to this.

A 2006 study by Wu et al. investigated the effects of docosahexaenoic acid (DHA), an omega-3 fatty acid, on estrogen metabolite levels in a randomized controlled trial of vegetarian women. The study found no changes in urinary 2-hydroxyestrone or 16a-hydroxyestrone levels in the six-week intervention (12).

In a 2011 randomized controlled trial by Young et al., 17 participants received diets that were of a high fat (40% of calories from fat), a low fat (20% of calories from fat), or a low fat with increased omega 3 fatty acid (23% calories from total fat, 3% from n-3 fatty acids) content for 8 weeks; given a 5 to 6 week washout period, then placed on the other diets (15). The study found a statistically significant association between the high fat diet and increased serum estradiol levels, with after 8 weeks of supplementation, a mean increase from 35.0 pmol/L to 52.8 pmol/L. However, no other statistically significant trend was found, indicating that the omega 3 supplementation did not serve as a protective effect (15). In a 2013 follow-up study, women were placed on a similar diet and cross-over pattern, and urinary estrogens were observed. In addition, urinary estrone levels were increased when the women were on the low fat dietary intervention (14).

Coburn et al. showed that serum and urine parent estrogens were correlated (54) so it is surprising that in the 2011 intervention, the high fat diet was associated with increased levels of serum estradiol (15) while in the 2013 intervention, the low-fat diet led to increased levels of urinary estrone (14). This may be explained by the small sample sizes in both interventions conducted by Young and colleagues; because the first only had 17 participants while the second had 46. Additionally, the results may vary due to hormone analysis differences; where the earlier study used radioimmunoassay (RIA), while the latter used liquid chromatography-tandem mass spectrometry (LC-MS/MS). While LC-MS/MS and RIA should generally be in agreement, the LC-MS/MS technique is known to be more sensitive and selective for measuring estrogen metabolites (55). However, as the differences found were in parent estrogens, this may not offer a strong explanation. Lastly, this may present a question about estrogen excretion with dietary change. A study measuring fat intake and urinary and serum estrogens may give more information.

Overall, the studies on fat intake and estrogen levels have been inconclusive. Nagata et al. and Young et al. both suggested that increasing fat intake was associated with increasing serum estrone and urine estradiol levels in 2011, respectively. While in 2013, Young et al. reported an inverse association between fat intake and urinary estrone levels.

#### Polyphenols (Green Tea, Grapeseed, Pomegranate)

Some *in vitro* studies have suggested that polyphenols and their catechins such as epigallocatechin-3-gallate (EGCG) can decrease the activity of the aromatase enzyme and affect development of mammary cells into cancer (56, 57). Aromatase inhibitors are frequently used in breast cancer treatment. The prospective use of nutraceuticals has sparked numerous more recent studies into various compounds that may decrease estrogen levels by modulation of the aromatase enzyme. In a study by Samavat et al., 937 women were asked to consume 4 capsules daily of Green Tea Extract (GTE) Catechin Complex, containing 843 mg of epigallocatechin-3-gallate (EGCG), equivalent to 5 cups of green tea intake per day (23). After one year of supplementation, the increased estradiol levels in the intervention group were statistically significant, while estrone, testosterone, SHBG, and androstenedione were unaffected (23). In a 2014 pilot study by Wahner-Roedler et al., 39 women consumed 200, 400, 600, or 800 mg of grapeseed extract, a good source of polyphenols, per day for 12 weeks; however, the study found no effect on serum estrogens, testosterone, or androstenedione (20). Kapoor et al. investigated another source of polyphenols, through pomegranate supplementation, on estrogen levels. Here, participants in the intervention arm were asked to consume 4 oz pomegranate juice twice daily for 3 weeks. The study found that when stratified by BMI, women in the normal weight category (< 25) had statistically significant decreases in estrone and testosterone levels, however, women with a BMI above 25 did not experience a change in serum sex hormone levels (27). While the compounds being studied have shown promising results *in vitro*, the results have not been confirmed in *in vivo* studies.

#### Soy

Soy is a source of another group of compounds called isoflavones, such as genistein, which have been shown to shrink and reduce the recruitment of blood vessels to growing tumors (58). There is variation between breast cancer incidence in western countries and East Asia. Some theories accredit this difference to specificities in diet and lifestyle factors, such as soy intake which is known to be much higher in Asian countries (58). In 2000, Pino et al. performed a 10-week intervention in which 20 women followed their normal diet but were supplemented with 30g of powdered soy milk every day. At the end of the study period, there was a statistically significant increase in SHBG levels in women with low baseline levels, below 55 nM/L (33). In women with circulating phytoestrogen levels greater than 0.6 mM/L, there was on average a 30% increase in SHBG, with an average of a 15.6% increase in SHBG in all women (33). Another 2000 study by Xu et al. found that the supplementation of a low-isoflavone diet containing 65 +/- 11 mg of isoflavones per day for 93 days led to an increase of the 2/16α-hydroxyestrone ratio. However, the high-isoflavone diet characterized by 132 +/- 22 mg of isoflavones per day did not have this same effect (29). A 2002 study by Persky et al. investigated the effect of varying levels of soy supplementation in 74 women over 24 weeks. Participants followed the Cholesterol Education Program Step 1 Diet, with one arm receiving 90mg of isoflavones from isolated soy protein (ISP90), 56mg of isoflavones from isolated soy protein (ISP56), or a control group receiving no supplementation (34). There was a borderline significant increase in SHBG in the ISP90 group, however there were no clear effects of the supplementation on the endogenous estrogens (34).

In contrast, a 2002 study by Wu et al. found that in 144 women in Singapore, there was a 15% reduction of estrone in women within the highest quartile of soy consumption, but this finding was not statistically significant. The study also found that women who had a soy intake greater than the median intake of soy had lower estrone levels than those that had an intake below the median (30).

In 2005, Wu et al. conducted an 8-week intervention of soy supplementation within a controlled diet on 57 postmenopausal women. Participants were placed on a very low-fat diet containing 11% of calories from fat, a low-fat diet with soy supplementation of 25% of calories from fat with 50mg of isoflavones per day, or a low-fat diet of 27% of calories from fat with no soy supplementation. The study found no change in endogenous estrogen levels, however there was a statistically significant decrease in testosterone in the low-fat diet treatment group receiving no soy supplementation (32). Sapbamrer et al. also investigated the effects of 30mg per day of soy isoflavone supplementation in 60 participants over 6 months. Likewise, the study found no change in estradiol in the experimental group, however there was a statistically significant decrease in estradiol in the control group (28). Lastly, a 2009 study by Furhman et al. found no significant trend in soy intake affecting overall estrone or estradiol levels in 167 women. There was however a statistically significant increase in 2-methoxyestrone levels (31).

#### Overall Dietary Pattern

The dominant diet in the United States referred to as a Western style diet, is composed of increasing intake of refined sugars and animal products, and decreased intake of whole grains and legumes. The Mediterranean style diet has been the topic of various research projects, investigating if it has a protective effect on breast cancer risk. In 2001, Berrino et al. investigated the effect of twice weekly cooking classes focusing on the Mediterranean style diet for 18 weeks. Of the participants in the intervention group, there was an average weight change of 4kg, and a decrease in the consumption of milk, cheese, meats, and saturated fat, and an increase in the consumption of whole grains, seeds, legumes, berries, and cruciferous vegetables (25). In addition, there was a statistically significant change in estradiol (-18.0%), testosterone (-19.5%), and SHBG (+25.2%) (25), however, when stratified for change in weight, the results were no longer statistically significant. A similar and later study by Carruba et al. also investigated the impact of cooking classes and adherence to the Mediterranean diet on estrogens in 106 women. Their study found that after 6 months, there was a reduction in total estrogens in the intervention group due to changes in metabolite levels including the average 80% decrease in 2-hydroxyestradiol (24). Additionally, there was an increase in urinary estradiol levels in the intervention group, possibly due to decreased metabolism. Sánchez-Zamorano et al. conducted a more recent cross-sectional study in 2016 on dietary patterns and estradiol levels, and found that women who adhered to a more Western diet, including increased intakes of red meat and eggs, had higher estradiol levels (26). Red meat and eggs are sources of dietary cholesterol, a precursor for endogenous estrogens, providing a possible explanation for why the Western diet may be associated with increased estrogen levels (26).

The Mediterranean diet and interventions studied focused on decreasing consumption of animal products, refined sugars, and added salt while increasing fish, legumes, nuts, seeds, whole grains, and vegetables. Some of these components may have protective effects in their chemical content, such as lignans and polyphenols, or their macronutrient content, such as specific types of fat intake.

### Exercise

#### Activity Level

Exercise is known to have physiological benefits such as maintaining a healthy weight, increasing cardiovascular fitness, and preventing type II diabetes mellitus. While exercise is a protective factor for many diseases, including breast cancer, its physiological effects in breast cancer prevention are not fully understood. Several cross-sectional studies have evaluated the overall activity level with endogenous estrogens and breast cancer risk. In 2007, Chan et al. investigated overall activity levels and endogenous estrogens in the EPIC-Norfolk cohort. A physical activity questionnaire was administered to cohort participants to determine overall activity levels at home, work, and recreationally, with metabolic equivalent of task (METs) being used to help stratify data. The study found that increased physical activity was associated with lower estradiol and testosterone levels and the testosterone to SHBG ratio when BMI and other covariates such as age, alcohol consumption, parity, and smoking status were accounted for (39). Similarly, in 2009 van Gils and colleagues investigated the association between activity levels and endogenous estrogens in the Prospect-EPIC cohort. This study used the Cambridge physical activity questionnaire and classified women into four quartiles of activity levels. After analysis, the investigators found that physical activity and free estradiol levels were inversely related when BMI was not adjusted for (43). However, after adjusting for BMI, DHEAS and physical activity were still positively related, and there was a significant association between androstenedione and cycling (43).

In 2016, Dallal et al. investigated the association between sedentary behaviors, measured by an accelerometer worn for one week, and endogenous estrogens in 542 participants. They found that increased physical activity was associated with overall lower levels of parent estrogens, even after adjusting BMI, with the highest tertials of sedentary behavior being associated with higher levels of total estrogens (40). Additionally, greater sedentary time was associated with an increase of the 16-pathway to parent estrogen ratio (40). In 2017, the effects of active and sedentary behaviors were further investigated by Oh et al. in 1804 women from the Women’s Health Initiative Observational Study. The study used an exercise frequency questionnaire to gauge normal activity levels and intensities. Over 10 hours of sedentary time per day was correlated with increased levels of unconjugated estrone and estradiol, with the relationship to unconjugated estrone remaining after adjusting BMI (42). Additionally, women who reported 15 MET-h of moderate to vigorous activity per week had lower estrone and estradiol levels than those who reported 0 MET-h per week of moderate to vigorous activity after accounting for age and other covariates (42). It should be noted that several studies used a questionnaire to gauge participant activity levels, which may introduce bias to the results.

#### Exercise Interventions

Existing research indicates that increased activity is associated with lower levels of endogenous estrogens, yet questions about the effect of exercise interventions on endogenous estrogen levels persist. In 2004, McTiernan et al. conducted a 12-month randomized controlled trial to investigate an exercise intervention of 45 minutes of moderate intensity exercise five days per week on 173 women. Women in the exercise intervention lost on average 1.4kg, had a decrease in estrone levels, and an increase in SHBG levels (41). Notably, the estrogens changed only in women who lost at least 0.5% body fat, and those who lost more weight saw more drastic changes (41). In 2004, Atkinson et al. investigated a similar 12-month exercise intervention, where in the first three months, women attended three exercise sessions at an exercise facility and two sessions on their own at home. For the final nine months, women attended one exercise session at the facility, and another four at home, working at 60-70% of their maximum heart rate for 45 minutes by week eight. At the end of the 12 months, participants had lost 1.3kg on average (44), similar to the weight loss seen in McTiernan’s study. However, there were no significant changes in urinary 2-hydroxyestrone or 16a-hydroxyestrone with the exercise intervention (44). Results by Friedenreich et al., who six years later in 2010, found after 12 months of an exercise intervention, where participants exercised at 70-80% of their maximum heart rate five days per week in a facility, there were statistically significant decreases in total estradiol, free estradiol, and SHBG (45). Women who adhered to 150-225 minutes per week of exercise had an average reduction of 18% serum estradiol levels than the controls. After adjusting for weight change and exercise adherence, the relationship between the intervention and total estradiol and free estradiol was still significant (45). Lastly, the study found that with increasing adherence to the protocol, the greater the change experienced (45).

More recently (2012), Matthews et al. also sought to understand the association between cardiovascular fitness and endogenous estrogen levels. After one year of intervention, where participants exercised at the more rigorous level of 70-80% of their maximum heart rate five days per week, participants in the intervention group saw a decrease in estradiol levels. Furthermore, after adjusting BMI, increased cardiovascular fitness was associated with lower 2-pathway estrogen metabolites (37).

#### Exercise Type and Dose

In 2015, Friedenreich followed up their 2010 study and investigated the relationship between the amount of exercise one participates in and endogenous estrogens. In the two intervention arms, women exercised for 30 or 60 minutes at 60-80% of their maximum heart rate five days per week for one year. Supervised sessions done at the facility were not only restricted to one modality. The exercise intervention was associated with decreased total estradiol, free estradiol, and estrone and increased SHBG (35). Obese women saw greater increases in SHBG in the high intensity 60-minute intervention, while non-obese women saw greater increases in SHBG in the moderate intensity 30-minute intervention; however, changes in hormone levels were similar in the two intervention groups (35). As the study notes, adherence to the protocol may have played a role in this discrepancy. In 2020, Gonzalo-Encabo conducted a randomized controlled trial of exercise intervention type and endogenous estrogens in overweight women. Women were randomized into an endurance arm, endurance + resistance (concurrent) arm, or a control arm of no training. Participants attended three sessions per week with a trainer for 12 weeks. Body mass and fat mass decreased in both the endurance and concurrent exercise groups, and women in the concurrent exercise group saw an increase in lean mass. Levels of total and free estradiol and SHBG did not change between the groups or controls (36). In participants that lost more than 2 kg, there was a statistically significant increase in SHBG (36).

### Diet and Exercise

With increasing evidence that both diet and exercise can influence endogenous estrogen levels, separately, several studies have used an intervention combining an exercise and dietary component, often involving weight loss as a metric. In 2000, Tymchuck et al. asked 22 participants to follow a very low fat (less than 10% of total calories) and high fiber (35-40g per 1000 calories) diet along with an exercise regimen of 30-60 minutes per day, 5 days per week, at an intensity of 70-85% of their maximum heart rate. The study found that women using hormone therapy lost 3.9kg while women not using hormone therapy lost 2.7kg over 3 weeks, with an increase in SHBG of 39% in the hormone replacement therapy group and the non-hormone replacement therapy group (38).

Campbell et al. in 2012 held a randomized controlled trial where participants were randomized into three arms based on intervention type: exercise only, diet only, or diet and exercise. Participants in the dietary intervention group met with a dietician several times throughout the study, attended group meetings, and kept food logs. Participants in the exercise intervention group attended supervised exercise sessions three days per week and did three at-home sessions. Women in the diet and exercise intervention group attended both levels of intervention for diet and exercise arms. After one year, the researchers found that estrone decreased in every arm, -9.6% in the diet only arm, -5.5% in the exercise only arm- and 11.1% in the diet + exercise arm (47). Estradiol changed in the diet only arm by -16.2%, and in the exercise + diet arm by -20.3%. On the other hand, SHBG increased in the diet only arm by +22.4% and in the exercise + diet arm by 25.8% (47). This may be explained by the average weight losses in each of the groups. Participants in the diet only intervention lost 9.1kg on average, participants in the exercise only intervention lost 2.8kg on average, and participants in the exercise and diet lost 9.8kg on average (47). Lastly, the study found that greater weight loss associated with greater metabolic changes. In another study by Carpenter et al., investigators measured the effects of a weight loss intervention on ductal fluid estrogens. For the diet portion of the intervention, the diet was composed of 7 servings of fruits and vegetables daily, 25-35 grams of fiber per day with 20% of calories from fat, 20% of calories from protein, and 50% of calories from carbohydrates (fiber content not specified), while achieving a 500-calorie deficit of the participant’s BMR. The participants also met with a trainer at least three times per week. At 12 weeks, there was a 24% reduction in estradiol levels in the ductal fluid (49).

Lastly, in a 2015 study by van Gemert and colleagues, where both interventions were associated with endogenous estrogens, participants were placed in a diet intervention or “mainly exercise” intervention or a control group (no intervention to current lifestyle patterns). Participants in the diet intervention had a caloric restriction of 3500 calories per week, while participants in the exercise intervention group had a caloric restriction of 4280 calories per day per week achieved by exercise and some dietary restriction. In the diet intervention group, participants met with dieticians, kept food logs, and were prescribed a weight loss plan. In the mainly exercise intervention group, participants attended four hours a week of a combined strength and endurance program. In the diet intervention, participants had an average percent weight loss of 6.09% compared to 6.87% in the mainly exercise arm. In the mainly exercise group, there were statistically significant decreases in testosterone and androstenedione. There were statistically significant increases in SHBG in both intervention arms, 12.6% in the diet arm and 19.0% in the mainly exercise arm, and decreases in total estradiol, -13.8% in the diet arm and -12.7% in the mainly exercise arm (48). However, after changes in body fat were accounted for, these changes attenuated. Across all studies, the average BMI of participants was in the borderline obese to obese range (33.6 in Carpenter et al., 30.9 in Campbell et al., 29.2 in van Gemert et al., and 32.1 in Tymchuck et al.). In all the studies reviewed, the weight loss interventions, whether exercise- or diet-based, resulted in a favorable change for endogenous estrogen levels.

#### Association Studies

As increased levels of endogenous estrogens are a risk factor for breast cancer incidence, it is important to understand the limitations of this model. Guinter et al. in 2018 derived an estrogen related dietary pattern based on the Prostate, Lung, Colorectal and Ovarian (PLCO) Cohort. The research team used reverse rank regression to find associations between foods listed in a food frequency questionnaire and estradiol levels in a subset of 653 postmenopausal women. The findings of the estrogen related dietary pattern (ERDP) were applied to 27488 women, who were broken into quartiles. Women in the highest quartile of the ERDP consumed the most refined grains (carbohydrate), tomatoes, cheese, and processed meats (11). Participants in the lowest quartile consumed the most coffee, nuts and seeds, fish, yogurt, and other vegetables (11). For 13 years, the study followed the PLCO Cohort to determine breast cancer incidence. In the highest quartile of the estrogen related dietary pattern, there were 481 diagnoses of breast cancer, accounting for 6.27% of the individuals in this category (11). In the lowest quartile of the estrogen related dietary pattern, there were 366 diagnoses of breast cancer, accounting for 5.25% of the individuals in this category (11). A step-wise but non-significant trend was seen, but it should be noted that physical activity was not evaluated. However, this team further investigated an estrogen related lifestyle score (ERLS), encompassing the estrogen related dietary pattern (ERDP), physical activity, weight status and alcohol use. This more comprehensive lifestyle score ranged from 0-6, with 0 having the lowest estrogenic potential and 6 having the greatest. They found that in 11.5 years of follow up within the PLCO Cohort, there was a greater incidence of breast cancer in individuals with a low ERLS. For example, in the group of participants with a score of 2 or lower, there were 459 breast cancer diagnoses, accounting for 6.1% of individuals in this category (59). In the group of participants with a score of 5 or greater there were 226 breast cancer diagnoses, accounting for 4.7% of individuals in this category (59). While both studies showed statistically significant associations between the ERDP or ERLS and breast cancer cases, when the entire PLCO cohort was surveyed, the ERLS had a stronger association with breast cancer incidence with a p-value <0.0001 (59). The ERDP measure had a p value<0.005 (11).

## Discussion

With the studies reviewed herein, it becomes clear that BMI plays an important role in regulating estrogen levels in the body. In a study by McTiernan et al. in 2012, researchers found that women with a high BMI in the highest quartile of physical activity had the lowest levels of estrogens while women in the lowest quartile of physical activity from the same BMI range had the highest, indicating that BMI may be an “effect measure modifier” (46). In many of the studies that included a weight loss intervention, reduction in body weight due to changes in diet and exercise had a subsequent decrease in overall estrogen levels, such as was seen in the study by van Gemert and colleagues. However, when the change in body fat was accounted for, these results were no longer significant (39, 41, 59). Thus, overall adiposity is important. The aromatase enzyme that catalyzes the reaction of androgens to endogenous estrogens is present in adipose tissue, so decreasing the amount of adipose tissue may decrease the production of the endogenous estrogens. While accounting for body fat and BMI nullified the statistical significance for some of the results in several studies, the finding presented here are still biologically relevant. If reducing excess body fat through dietary or exercise interventions decreases subsequent risk for breast cancer by lowering endogenous estrogens, it may be a useful and lower cost intervention for breast cancer prevention. Worthy of mention, there may be an interference between fat intake and the pathways in which estrogens are metabolized, as suggested by the Fowke et al. study. Thus, more data and especially that from larger cohort studies is needed to understand the relationship between fat intake and estrogen levels in postmenopausal women, particularly as regards to BMI.

For polyphenol-focused studies, variation in results may be due to the small sample size of the latter two studies discussed, both having less than 65 participants in either. Another explanation may be the dose relationship between the polyphenols and the subject. With increasing BMI, the dose provided in the studies may not be sufficient to modulate the effects of the increased aromatase activity (20, 27).

Regarding studies evaluating soy, results were mixed. The Sapbamrer et al. study may have had their results influenced by their study design; because the trial was not randomized, and the investigators chose women that preferred eating soy foods to be in the experimental group. These individuals may have already had high soy intake before starting the study, which may have interfered with the study results to demonstrate a modification. Additionally, the control group had a decrease in estradiol levels. As this study was conducted in Thailand, in a region where soy intake may be a normal part of the diet, asking women to avoid these foods may have caused the decrease in estradiol. As the investigators state, there may be a mechanism in which soy influences and maintains estrogen levels that needs to be investigated. Further, a systematic review in 2009 highlighted that there were no statistically significant associations between soy isoflavone supplementation and serum estrogens or SHBG (60). However, there was some evidence that SHBG may be influenced by soy isoflavones. The Pino et al. study found a significant increase in SHBG, especially in women who had low levels at baseline. Additionally, the study by Persky et al. found a borderline significant association between soy intake and SHBG (p = 0.06), indicating another potential avenue for research.

Another important insight into these studies is the sustainability of the interventions cited. The major difference between the Atkinson and Friedenreich studies is maintaining better control and presumably compliance of the level of exercise in a facility-based atmosphere, compared to entrusting participants to adhere to the level of desired rigor when exercising at home. Another slight difference, albeit likely not significant, is the 10% threshold increase for maximum heart rate achieved. While there may be some differences for participants at the 60% level in the Atkinson study compared to participants exercising at the 80% rate in the Friedenreich study, the mean between the two is the 70% maximum heart rate. Further, a limitation of the Matthews study compared to the others reviewed is that no information on where the physical activity was based (home *vs* facility *vs* mixed-model) was provided.

In the Berrino et al. study, a cooking class intervention was used to see how the dietary pattern would change. Women that attended the cooking classes did not have a controlled diet; however they still had favorable outcomes. The women in this group had an average weight loss of 4kg, decreased their consumption of milk, cheese, meats, and saturated fat, and increased their consumption of whole grains, seeds, legumes, berries, and cruciferous vegetables (25). This may be contrasted with the study by Tymchuck et al., where obese women were asked to consume a high fiber, low fat diet, and exercise 30-60 minutes daily five days per week (38). While both of these studies had favorable outcomes, if women are to sustain weight loss until a desired target is reached, careful thought needs to be put into the intervention from the start that incorporates a plan for long-term maintenance. Considerations on maintaining the intervention and/or benefits there-of, such as continued exercise habits and/or dietary changes, must include outcome goals that equate to a definitive lifestyle change. Women may feel that attending cooking classes and gradually changing their eating habits may be a more attainable than starting on a controlled diet and rigorous exercise regimen without much preparation. It can be quite overwhelming, and especially if in the context of a new breast cancer diagnosis. Thus, in addition to interventional strategies, psychosocial counseling should be considered to support participants and patients toward reducing cancer risk and achieving better overall health (61).

Overall, diet and exercise interventions have been shown to decrease endogenous estrogen levels, which may, in turn, reduce breast cancer risk. Some of the strongest associations included weight loss *via* diet and diet + exercise interventions, reducing alcohol consumption, and consuming a varied dietary pattern, similar to the Mediterranean diet. More research should be done on the effects of specific nutritional components on endogenous estrogen levels to understand the effect that the components have on their own and in combination within the diet.

## Author Contributions

AW, SS, and DS conceived of the review topic. AW and DS outlined the content for the review. AW performed the systematic literature search. JC performed fact checking for the included references. AW drafted the manuscript. All authors contributed to the article and approved the submitted version.

## Funding

The work was supported by the UNC-Chapel Hill Nutrition Research Institute (NRI) Faculty Development Program (DS); and the NRI Metabolomics and Exposure Laboratory (SS).

## Conflict of Interest

The authors declare that the research was conducted in the absence of any commercial or financial relationships that could be construed as a potential conflict of interest.

## Publisher’s Note

All claims expressed in this article are solely those of the authors and do not necessarily represent those of their affiliated organizations, or those of the publisher, the editors and the reviewers. Any product that may be evaluated in this article, or claim that may be made by its manufacturer, is not guaranteed or endorsed by the publisher.
